# Jejuketomycins A and B, polyketide glycosides with cancer cell migration inhibitory activity from *Streptomyces* sp. KCB15JA151†

**DOI:** 10.1039/d2ra04039e

**Published:** 2022-08-10

**Authors:** Jun-Pil Jang, Gil Soo Kim, Tae Hoon Oh, Beomcheol Park, Minhee Kim, Gwi Ja Hwang, Hyeok-Won Lee, Jin-Gyeom Lee, Young-Soo Hong, Jong Seog Ahn, Sung-Kyun Ko, Jae-Hyuk Jang

**Affiliations:** Chemical Biology Research Center, Korea Research Institute of Bioscience and Biotechnology Cheongju 28116 Korea jangjh@kribb.re.kr; Department of Biomolecular Science, KRIBB School of Bioscience, University of Science and Technology Daejeon 34113 Korea; Central Research and Development, HanpoongPharm. Co., LTD. Wanju 54843 Korea; College of Pharmacy, Chungbuk National University Cheongju 28160 Korea; Biotechnology Process Engineering Center, Korea Research Institute of Bioscience and Biotechnology Cheongju 28116 Korea

## Abstract

Two new polyketide glycosides jejuketomycins A (1) and B (2), were isolated from a culture of *Streptomyces* sp. KCB15JA151. Their chemical structures including the absolute configurations were determined by detailed analyses of the NMR and HRMS data and ECD calculations and spectral data. Compounds 1 and 2 possess an unusual 6/6/8 tricyclic ring system. Biological evaluation with the wound healing assay and time-lapse cell tracking analysis revealed that compounds 1 and 2 have significant inhibitory activities against cancer cell migration with low cytotoxicity.

## Abstract 2

Two new polyketide glycosides jejuketomycins A (1) and B (2), were isolated from a culture of *Streptomyces* sp. KCB15JA151.

## Graphical abstract

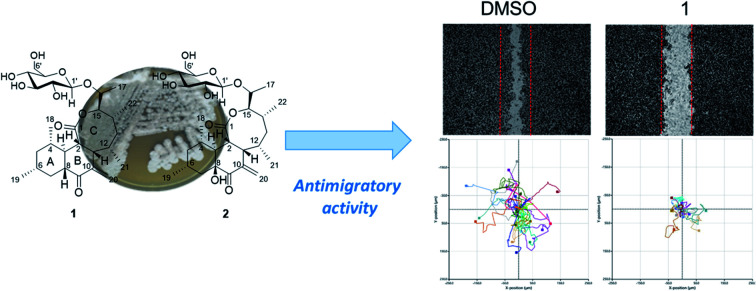

## Introduction

Microbial secondary metabolites provide various chemical templates for clinically useful lead compounds in the pharmaceutical industry.^1,2^ Actinomycetes, especially those from the genus *Streptomyces*, are known to be a prolific source of bioactive secondary metabolites. They produce a wide variety of products including polyketides, peptides, terpenes, and glycosides.^3–6^ Among them, polyketide natural products exhibit a range of functional and structural diversity and medicinally important activities, including antibiotic, anticancer, antifungal, and antiparasitic.^7,8^ Chemical studies and genome sequence analysis of many strains of actinomycetes have revealed the presence of biosynthetic gene clusters whose number far exceeds the number of known secondary metabolites produced by those bacteria. Usually, only a small number of the biosynthetic gene clusters are expressed under typical laboratory culture conditions to produce detectable amounts of compounds.^9^ This information further underscores the enormous potential of actinomycetes as a source of bioactive natural products.

**Fig. 1:**
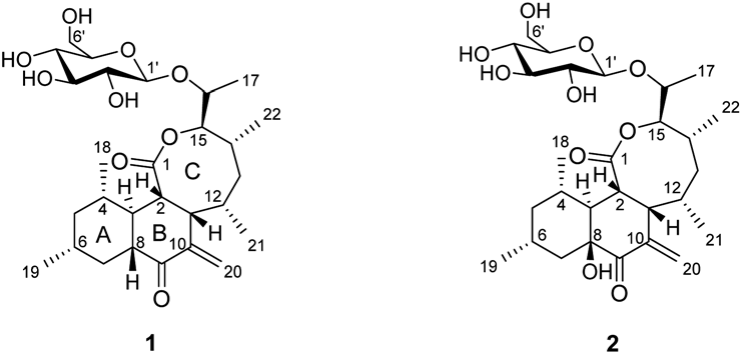
The chemical structures of 1 and 2.

Cell migration is involved in a number of physiological processes including ovulation, embryonic development, tissue regeneration (wound healing), and inflammation. These migration activities of cells *in vitro* are thought to be related to many *in vivo* cellular behaviors such as tumor angiogenesis and metastasis.^10^ Thus, cell migration is attracting much attention as one of the alternative strategies for the development of anti-cancer chemotherapies. Several natural products such as withaferin A,^11^ prodigiosin,^12^ and migrastatin^13^ have been reported to inhibit cell migration. As part of our continuing efforts to find inhibitors of cell migration, *Streptomyces* sp. KCB15JA151, a soil-derived bacteria, caught our interest and was subjected to a systematic chemical investigation, resulting in the isolation and identification of two new polyketide glycosides, jejuketomycins A (1) and B (2) (Fig. 1). Herein, the isolation, structure elucidation, and bioactivity evaluation of these compounds are reported.

## Results and discussion

*Streptomyces* sp. KCB15JA151 were cultured in PDB media for 7 days at 28 °C, and the broth and mycelia extracts were partitioned with EtOAc. The EtOAc extracts were purified by ODS vacuum flash chromatography and reversed-phase HPLC to yield jejuketomycins A (1) and B (2). Jejuketomycin A (1) was isolated as a yellowish powder and its molecular formula was established as C_28_H_44_O_9_ based on the HRESIMS and NMR data. The ^1^H NMR spectrum (DMSO-*d*_6_) exhibited two *exo*-methylene protons (*δ*_H_ 6.15 and 5.27), six oxygen-bearing methine protons (*δ*_H_ 4.46, 4.07, 3.15, 3.11, 3.08, and 2.97), an anomeric proton (*δ*_H_ 4.17), two oxygen-bearing methylene protons (*δ*_H_ 3.68 and 3.44), eight methine protons (*δ*_H_ 3.33, 2.82, 2.57, 2.24, 2.23, 1.46, 1.41, and 1.32), six methylene protons (*δ*_H_ 1.91, 1.67, 1.57, 1.10, 0.80, and 0.60), and five methyl protons (*δ*_H_ 1.12, 1.08, 0.90, 0.87, and 0.75).

The ^13^C NMR was combined with HSQC-DEPT data, which indicated the existence of 28 carbons, including two carbonyl carbons (*δ*_C_ 201.1 and 177.9), a non-protonated sp^2^ carbon (*δ*_C_ 140.5), an *exo*-methylene carbon (*δ*_C_ 122.0), an anomeric carbon (*δ*_C_ 101.6), six oxygen-bearing methine carbon (*δ*_C_ 86.2, 77.6, 77.3, 73.6, 71.0, and 70.7), an oxygen-bearing methylene carbon (*δ*_C_ 61.8), eight methine carbons (*δ*_C_ 50.1, 46.7, 45.4, 41.5, 39.3, 37.9, 35.6, and 30.1), three methylene carbons (*δ*_C_ 43.7, 38.1, and 34.7), and five methyl carbons (*δ*_C_ 24.8, 22.9, 19.5, 18.7, and 16.7) (Table 1).

**Table 1:** ^1^H and ^13^C NMR spectroscopic data for 1 and 2 in DMSO-*d*_6_

Position	1		2	
	*δ*_C_	*δ*_H_, mult (*J* in Hz)	*δ*_C_	*δ*_H_, mult (*J* in Hz)
1	177.9		178.0	
2	50.1	2.82, t (8.2)	48.6	2.68, t (8.2)
3	41.5	1.32, ddd (13.4, 10.4, 7.7)	44.9	1.48, q (10.9, 7.7)
4	39.3	1.46, m	33.5	1.79, m
5	43.7	0.60, q (12.2), ax	43.8	0.61, q (12.2), ax
		1.57, dq (13.4, 2.1), eq		1.58, dq (13.2, 2.1), eq
6	30.6	1.41, m	26.4	1.81, m
7	34.7	0.80, q (12.8), ax	41.0	0.96, t (12.9), ax
		1.91, dq (13.6, 2.1), eq		1.90, d (13.6), eq
8	45.7	2.57, ddd (13.7, 11.3, 3.7)	73.7	
9	201.1		198.7	
10	140.5		140.1	
11	46.7	3.33, m	45.8	3.96, m
12	35.6	2.33, m	35.6	2.33, m
13	38.1	1.10, ovl^a^, ax	37.8	1.08, ovl^a^, ax
		1.67, ddd (14.9, 7.2, 3.4), eq		1.66, dt (14.9, 3.4), eq
14	37.9	2.24, m	37.9	2.24, m
15	86.2	4.46, dd (10.4, 1.9)	86.1	4.49, dd (10.4, 1.9)
16	71.0	4.07, qd (6.4, 2.0)	71.1	4.07, qd (6.4, 2.0)
17	16.7	1.08, d (6.4)	16.7	1.08, d (6.4)
18	19.5	0.75, d (6.5)	19.2	0.74, d (6.5)
19	22.9	0.90, d (6.5)	22.6	0.88, d (6.4)
20	122.0	6.15, br s	123.5	6.18, br s
		5.27, br s		5.36, br s
21	24.8	1.12, d (7.6)	25.0	1.12, d (7.6)
22	18.7	0.87, d (6.7)	18.7	0.87, d (6.4)
1′	101.6	4.17, d (7.9)	101.6	4.16, d (7.9)
2′	73.6	2.97, t (8.2)	73.6	2.98, t (8.2)
3′	77.6	3.15, t (8.8)	77.6	3.16, t (8.8)
4′	70.7	3.08, dd (10.6, 7.7)	70.7	3.09, dd (10.6, 7.7)
5′	77.3	3.11, ddd (9.2, 5.8, 2.1)	77.3	3.11, ddd (9.2, 5.8, 2.1)
6′	61.8	3.44, dd (10.3, 5.3)	61.8	3.44, dd (10.3, 5.3)
		3.68, d (11.2)		3.68, d (11.2)
8-OH				5.47, br s

a Overlapped signal.

**Fig. 2:**
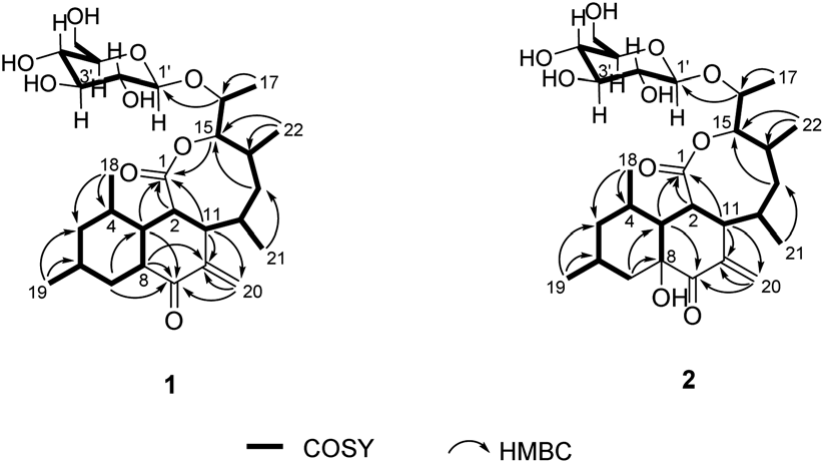
Key 2D NMR correlations of 1 and 2.

The planar structure of 1 was determined by extensive 2D NMR analysis (Fig. 2). The COSY spectrum of 1 showed two spin systems. The large spin system was used in the structural elucidation of aglycone, and the small one was used in sugar unit. Ring A was established by the COSY correlation of H-3/H-4/H-5/H-6/H-7/H-8. The methyl substitutions on ring A were also determined by the COSY correlation from H-18 to H-4 and from H-19 to H-6. Ring B was determined by the COSY correlations of H-8/H-3/H-2/H-11 with the HMBC correlations from H-3 to C-9, H-8 to C-10, H-11 to C-10 and C-20, and H-20 to C-10. The COSY correlations of H-2/H-11/H-12/H-13/H-14/H-15 with the HMBC correlations of H-2 to C-1, H-11 to C-1, and H-15 to C-1 constructed ring C, which showed an eight-membered lactone. The COSY correlations of H-12/H-21, H-14/H-22 and H-15/H-16/H-17 suggested that hydroxyethyl and methyl groups were attached to ring C.

An anomeric carbon (*δ*_C_ 101.6) indicated the existence of a sugar unit. The sugar unit was deduced by the COSY correlation from H-1′ to H-6′ with the HMBC correlation from H-5′ to C-1′. The HMBC correlation from H-16 to C-1′ suggested that the sugar unit was bound with the aglycone *via* an *O*-glycosidic linkage. The result of the NMR analysis suggested that the planar structure of compound 1 was very similar to satosporin B^14^ except for the *exo*-methylene. Therefore, compound 1 was determined to be a new polyketide glycoside and named as jejuketomycin A.

**Fig. 3:**
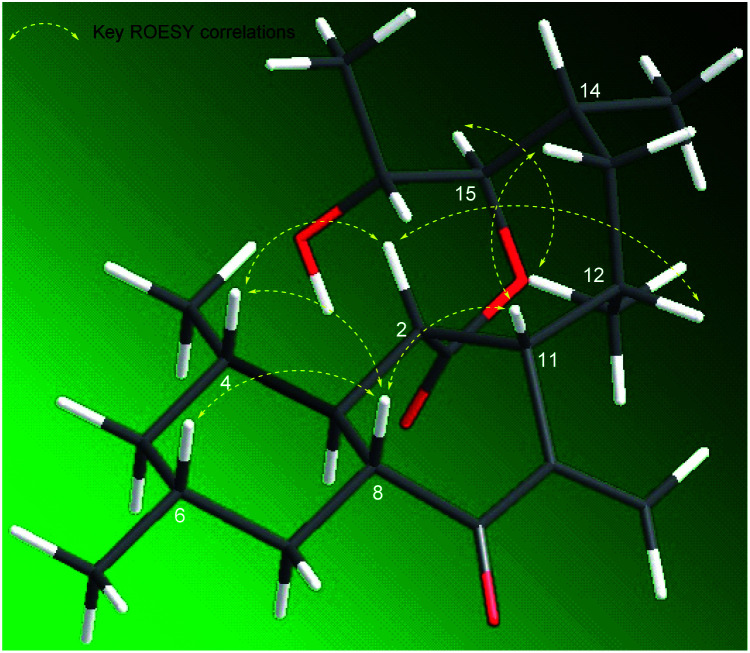
Key ROESY correlations of 1.

The ROESY spectrum and coupling constants were obtained to determine the relative configuration of compound 1 (Table 1 and Fig. 3). The large coupling constant of the anomeric proton (*δ*_H_ 4.17, *J* = 7.6 Hz) suggested a β-glycosidic linkage. The other protons in the sugar unit also demonstrated a large coupling constant ranged from 8.2 to 11.2 Hz, which suggested that all of the methine protons were located on axial positions. The ROESY correlation of H-1′ to H-3′, H-1′ to H-5′, and H-2′ to H-4′ also supported the axial positions of the methine protons. Thus, the sugar unit in compound 1 was established β-glucose. The large coupling constants of H-3 (*δ*_H_ 1.32, *J* = 13.4, 7.7, 10.4 Hz), H-5_ax_ (*δ*_H_ 0.60, *J* = 12.2 Hz), H-7_ax_ (*δ*_H_ 0.80, *J* = 12.8 Hz), H-8 (*δ*_H_ 2.57, *J* = 13.7, 11.3, 3.7 Hz) suggested that H-3, H-4, H-5_ax_, H-6, H-7_ax_, and H-8 occupied axial positions. The ROESY correlations of H-2 to H-4, H-2 to H-8, H-4 to H-8, H-6 to H-8, H-8 to H-11, and H-11 to H-13 implied that H-2, H-4, H-6, H-8, H-11, and H-13_ax_ were on the same side. The rigid structure by the *exo*-methylene and the ROESY correlation between H-8/H-11 and H-20/H_3_-21 with no correlation observed between H-11/H_3_-21 indicated H_3_-21 was on the opposite axial position. The ROESY correlations H-12 to H-13_ax_ and H-13_ax_ to H-14 in the 1, 2-axial equatorial interactions suggested H-12 and H-14 occupied equatorial positions. The large coupling constant of H-15 (*δ*_H_ 4.46, *J* = 10.4, 1.9 Hz) and the ROESY correlation between H-15 with H_3_-21 implied that H-15 was placed on the axial position. The coupling constant of H-16 (*δ*_H_ 4.07, *J* = 6.4, 2.0 Hz) and the ROESY correlation between H-15/H-16, H-15/H_3_-17, and H-16/H_3_-22 were very similar to those of satosporin A–C,^14^ which suggested an anti-configuration of the hydroxy groups between C-15 and C-16. Therefore, the relative configuration of aglycone in compound 1 was proposed to be 2S*, 3R*, 4S*, 6R*, 8R*, 11S*, 12S*, 14R*, 15R*, 16R*.

The time-dependent density functional theory-electronic circular dichroism (TDDFT-ECD) calculation was conducted to established the absolute configuration of compound 1.^14^ The aglycone structure was used to calculating the ECD spectrum to save on the computational time.^15^ All conformers of aglycone were geometry optimized using the B3LYP/6-311G(d) basis set.

**Fig. 4:**
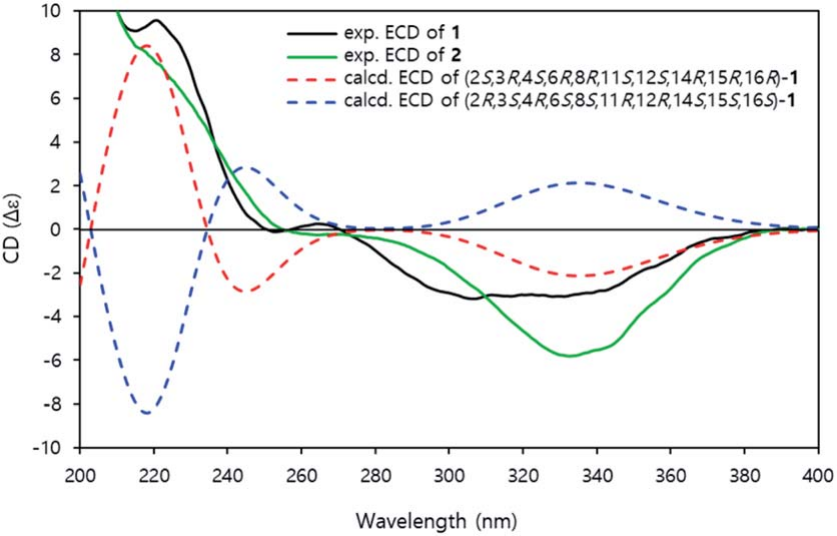
Experimental ECD and calculated ECD spectra of 1 and 2.

The ECD spectra were also calculated using the B3LYP/6-311G (d) basis set from all of the optimized conformers. The experimental ECD spectra of 1 showed positive cotton effects at 220 nm and negative cotton effects at 310–340 nm. The calculated ECD curve of (2*S*, 3*R*, 4*S*, 6*R*, 8*R*, 11*S*, 12*S*, 14*R*, 15*R*, 16*R*)-1 demonstrated a similar pattern of the experimental ECD spectra (Fig. 4). Therefore, the absolute configuration of 1 was established as 3*R*, 4*S*, 6*R*, 8*R*, 11*S*, 12*S*, 14*R*, 15*R*, 16*R*.

Jejuketomycin B (2) was isolated as a yellowish powder. The molecular formula was established as C_28_H_44_O_10_ on the basis of the HRESIMS data. The molecular formula showed the presence of a hydroxy group, compared to 1, which indicates that 2 is an analogue of 1 with slight modifications. A comparison of the 1D NMR data (Table 1) of compounds 1 and 2 revealed that their planar structures were very similar, except for the absence of the signal of the OH proton (8-OH, *δ*_H_ 5.10) in 2 (Fig. 2). The identical ROESY correlations and the experimental ECD spectra, closely similar the ^1^H and ^13^C NMR data, and the presumed same biosynthetic pathway led to the conclusion that the configuration of 2 was the same as that of 1. Thus, compound 2 was named as jejuketomycin B.

**Fig. 5:**
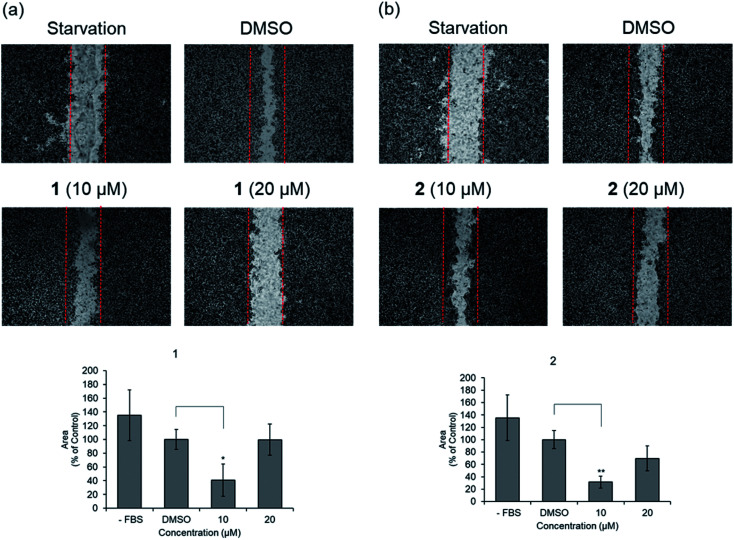
Effects of scratched wound healing in MDA-MB-231 cells for (a) compound 1. (b) Compound 2.

**Fig. 6:**
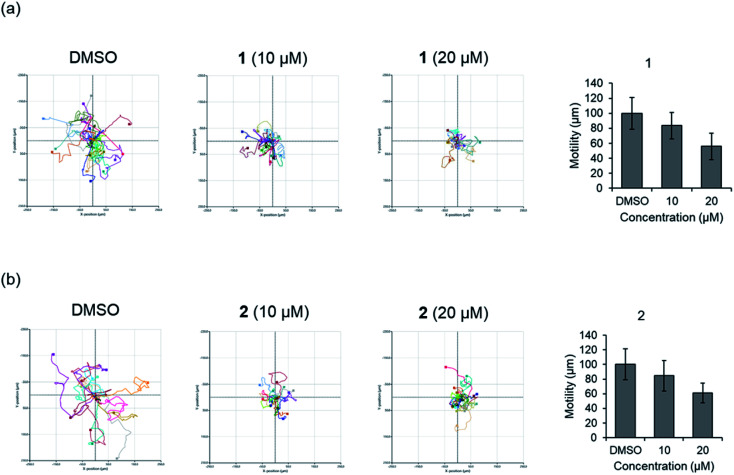
Time-lapse cell tracking analysis of cellular movement for (a) compound 1. (b) Compound 2.

Before evaluating the cancer cell migration inhibitory activity of 1 and 2, their cytotoxic effects were tested against MDA-MB-231, PC12, MEF, A549, and HeLa cells. As shown in Fig. S16,†1 and 2 did not show any cytotoxicity up to 50 μM, whereas a slightly decreased cell number was observed in the cells treated with 100 μM. The inhibitory effect of 1 and 2 on the migration of MDA-MB-231 cells with the scratched wound healing assay was determined (Fig. 5). After treatment with the indicated concentrations of 1 and 2 for 24 h, the rate of cells migrating to the scratch wound healing area was significantly decreased 1 (100 ± 22% inhibition) and 2 (69.8 ± 19.9% inhibition). To further evaluate the effect of 1 and 2 on cell migration, time-lapse cell tracking analysis was performed by a HoloMonitor M4 time-lapse cytometer. After treating the cells with 1 and 2, they were captured every 15 min for 12 hour. The migration and motility of 1 and 2 treated cells were remarkably decreased in a dose-dependent manner (Fig. 6).

## Experimental

### General experimental procedures

The specific rotations were measured on a JASCO P-1020 polarimeter that uses a 100 mm glass microcell. UV spectra were obtained on a NanoDrop 2000 spectrophotometer and UltiMate DAD-3000 connected to a Thermo Scientific Dionex Ultimate 3000 Rapid Separation LC system (UPLC-PDA) using a Waters HSS T3 column (Waters, 2.1 × 150 mm, 2.5 μm). IR spectra were recorded on a Bruker VERTEX 80v FT-IR spectrometer. The NMR spectra were recorded on Bruker AVANCE HD 700 and 800 NMR spectrometers at the Korea Basic Science Institute (KBSI) in Ochang, South Korea. Chemical shifts were referenced to a residual solvent signal (DMSO-d6 *δ*_H_ 2.50, *δ*_C_ 39.51). High-resolution electrospray ionization mass spectrometry (HRESIMS) data were acquired with a Q-TOF mass spectrometer on a SYNAPT G2 (Waters) at KBSI in Ochang, South Korea. The CD spectra were obtained on a JASCO J-1500 circular dichroism spectrophotometer at KBSI in Ochang, South Korea. Liquid chromatography-mass spectrometry (LC-MS) was performed with a Thermo LTQ XL linear ion trap attached to an ESI source that was connected to a Thermo Scientific Dionex Ultimate 3000 Rapid Separation LC system (ESI-LC-MS) using a Waters HSS T3 column (Waters, 2.1 × 150 mm, 2.5 μm). Open column chromatography was performed with a silica gel (Merck, silica gel 60 (0.063–0.200 mm)). Vacuum liquid column chromatography was carried out with an ODS (Cosmosil, 75 μm). Semi preparative C18 (Cosmosil 5C18-MS-II, 5 μm, 10 × 250 mm) columns were used for HPLC on a YL9100 HPLC system equipped with a photodiode array detector (YL9160) that uses HPLC grade solvents (Burdick & Jackson).

### Microorganism collection and identification

The strain KCB15JA151 was isolated from a soil sample from an agricultural field of garlic on Jeju Island, Korea. A soil sample was spread onto selective medium (24.0 g of Potato Dextrose Broth and 17 g of agar per 1 L of distilled water, pH 5.6) supplemented with Chloramphenicol (50 ppm) and Kanamycin (100 ppm). Strain KCB15JA151 was isolated from the selective media and transferred onto potato dextrose agar (PDA). Strain KCB15JA151 (GenBank accession no. MW282889) was identified as *Streptomyces* sp. by 16S rRNA gene sequencing, which showed a 99.7% similarity to *Streptomyces roietensis* WES2.

### Fermentation, extraction, and isolation

The *Streptomyces* sp. KCB15JA151 was grown in potato dextrose broth (PDB) for 7 days at 28 °C on a rotary shaker operating at 165 rpm in a baffled Erlenmeyer flask. The cultured broth (45 L) was divided into the filtered broth and mycelia by Büchner funnel. The filtered broth was extracted with an equal volume of ethyl acetate three times. The mycelia were extracted with an equal volume of 80% acetone in water followed by evaporation under reduced pressure to remove the organic solvent. The aqueous solution of the acetone extract was extracted three times with an equal volume of ethyl acetate. The combined extracts (14.49 g) from the supernatant and mycelia were fractionated by silica gel column chromatography (70 i.d. × 320 mm) and eluted with a stepwise gradient of CHCl_3_–MeOH (50 : 0, 30 : 1, 20 : 1, 10 : 1, 7 : 1, 5 : 1, 3 : 1, 2 : 1, 1 : 1, and 0 : 100 (v/v)). The bioactive fraction 4 (CHCl_3_–MeOH, 10 : 1, 1.25 g) was separated by reversed-phase C_18_ vacuum liquid column chromatography (50 i.d. × 250 mm) eluted with a stepwise gradient of MeOH in water (20%, 30%, 40%, 50%, 60%, 70%, 80%, 90%, and 100%). The active fractions 4–5 (60% MeOH, 95.2 mg) were purified by reversed phase HPLC (Cosmosil 5C_18_-MS-II, 5 μm, 10 × 250 mm) that used a linear gradient condition (35–70% MeCN–H_2_O containing 0.05% formic acid, flow rate 3 mL min^−1^) to yield compounds 1 (8.7 mg) and 2 (2.7 mg).

### Characterization of compounds 1 and 2

#### Jejuketomycin A (1)

Yellowish powder; [*α*]^25^_D_ +1.5 (*c* 0.05, MeOH); UV (MeOH) *λ*_max_ (log *ε*) 220 (4.30) nm; CD (MeOH) Δ*ε* 220 (9.56), 264 (0.25) 306 (−3.18); IR (ATR) *ν*_max_ 3376, 2917, 1729, 1377, 1070, 1023, 596 cm^−1^; ^1^H and ^13^C NMR spectroscopic data, see Table 1; HRESIMS *m*/*z* 547.2882 [M + Na]^+^ (calcd for C_28_H_44_O_9_Na, 547.2883).

#### Jejuketomycin B (2)

Yellowish powder; [*α*]^25^_D_ +2.3 (*c* 0.05, MeOH); UV (MeOH) *λ*_max_ (log *ε*) 220 (4.23) nm; CD (MeOH) Δ*ε* 220 (7.95), 330 (−5.98); IR (ATR) *ν*_max_ 3439, 2916, 1729, 1606, 1375,1062, 531 cm^−1^; ^1^H and ^13^C NMR spectroscopic data, see Table 1; HRESIMS *m*/*z* 539.2851 [M − H]^−^ (calcd for C_28_H_43_O_10_, 539.2861).

### ECD calculation

The conformational search was conducted using Avogadro 1.2.0 with the MMFF94 force field.^16^ The geometry optimization of all conformers were calculated using Gaussian 09 with the B3LYP/6-311g(d) basis set. TD-DFT method was performed at the B3LYP/6-311g(d) level of theory using Gaussian 09 to obtain the calculated ECD spectrum of each optimized conformers. The Boltzmann-weighted ECD spectrum was generated from each calculated spectrum using SpecDis 1.71.

### Cell culture

PC12 (pheochromocytoma of rat adrenal medulla), HeLa (Human cervix cancer), A549 (Human lung cancer), MEF (Mouse Embryonic Fibroblasts), and MDA-MB-231 (Human breast cancer) cells were purchased from the American Type Culture Collection (ATCC). The cells were routinely cultured in DMEM (Dulbecco's modified Eagle's medium; Welgene, Korea, LM 001-05) containing 10% FBS (fetal bovine serum; Welgene, Korea, S001-07), 100 units penicillin, and 100 μg mL^−1^ streptomycin (Gibco, USA, 15140-122) at 37 °C in humidified atmosphere containing 5% CO_2_.

### Cell viability assay

Cell viability was determined using the EZ-Cytox colorimetric assay (Daeil Lab service, 0793). For the cytotoxicity, PC12, HeLa A549, MEF, and MDA-MB-231 cells were cultured in 96-well plates (1.0 × 10^4^ cells per well) for 12 h. The cells were treated with test compounds at the indicated concentrations for 24 h. After the treatment for 24 h, media were replaced with EZ-Cytox solution and incubated with the cells for 1 h. Live cells were read in a microplate reader (Molecular Devices, Spectra Max 190) at 450 nm. Cell viability was normalized to the control group.

### Scratch wound healing assay

MDA-MB-231 cells were cultured in 24-well plates (9.0 × 10^4^ cells per well) for 12 h. Each well was artificially wounded by creating a scratch in the center with a 200 μL tip and cultivated in DMEM with 10 μg mL^−1^ mitomycin C for 3 h. The cells were treated with the test compounds at the indicated concentrations for 24 h. Cells were fixed in 4% paraformaldehyde for 12 min, stained with 0.2% crystal violet, captured by a microscope and analyzed with the ZEN software (ZEISS, Germany). The wound closure areas were measured with ImageJ (Software 1.48q, Rayne Rasband, National Institutes of Health, USA).

### Time-lapse cell tracking analysis

MDA-MB-231 cells (9 × 10^3^ cells/100 μL) were seeded in μ-Slide I(ibidi, Germany, 80106). After 3 h, 900 μL of DMEM were added and incubated overnight. The test compounds with the indicated concentrations were treated and directly transferred to the HoloMonitor M4 time-lapse cytometer (Phase Holographic Imaging) kept in a 37 °C incubator. The live cells were captured every 15 min for 12 h, and the migration was analyzed with the HoloStudio M4 software.

### Statistics

Data are presented as the mean ± standard error of the mean (SEM) of at least three independent experiments (*n* = 3). Statistical analyses were performed using GraphPad Prism (GraphPad Software, 9.4.0, San Diego, CA, USA). The differences were considered statistically significant at *p < 0.05 and, **p < 0.01, when compared to the DMSO control group.

## Conclusions

Jejuketomycins A (1) and B (2) are structurally unique glycosylated polyketides with an unusual 6/6/8 tricyclic ring system. Satosporin produced by Kitasatospora griseola is the most similar compound with a 6/6/8 tricyclic ring. However, the jejuketomycins exhibit a different *exo*-methylene group at the C-10 position. In previous reports, it was estimated that satosporins are likely biosynthesized by a type I polyketide synthase using d-lactate as an unusual starting unit.^14^ However, we were unable to identify candidate biosynthetic genes matching with the proposed satosporine polyketide assembly in the draft genome sequencing data of *Streptomyces* sp. KCB15JA151. Therefore, these new satosporin derivatives with the *exo*-methylene residue are expected to be synthesized by a rare biosynthetic machinery.^17^ In particular, the mechanism of forming tricyclic rings with the *exo*-methylene residues will be an interesting topic. The discovery of the jejuketomycins expands the diversity of the structural and biological aspects of polyketide natural products and also demonstrates that actinobacteria may have significant potential in the biosynthesis of bioactive organic molecules with unique skeletal frameworks.

## Conflicts of interest

There are no conflicts to declare.

## Supplementary Material

RA-012-D2RA04039E-s001
