# Supplementary material for: Jejuketomycins A and B, polyketide glycosides with cancer cell migration inhibitory activity from Streptomyces sp. KCB15JA151
Source: RSC Adv. 2022 Aug 10;12(35):22360–6. doi: 10.1039/d2ra04039e (PMC9364360; doi:10.1039/d2ra04039e)
Supplement: RA-012-D2RA04039E-s001 [file RA-012-D2RA04039E-s001.pdf]

Electronic Supplementary Material (ESI) for RSC Advances.
This journal is © The Royal Society of Chemistry 2022

Supplementary data

Jejuketomycins A and B, Polyketide Glycosides with Cancer Cell Migration Inhibitory Activity from *Streptomyces* sp. KCB15JA151

Jun-Pil Jang [‡a], *Gil Soo Kim* [‡c], *Tae Hoon Oh* [‡ad], *Beomcheol Park* [ad], *Hyeok-Won Lee* [e], *Jin-Gyeom Lee* [e], *Sung-Kyun Ko* [ab], *Young-Soo Hong* [ab], *Jong Seog Ahn* [ab], *and Jae-Hyuk Jang* [*ab]

[a] Chemical Biology Research Center, Korea Research Institute of Bioscience and Biotechnology, Cheongju 28116, Korea

[b] Department of Biomolecular Science, KRIBB School of Bioscience, Korea University of Science and Technology, Daejeon 34113, Korea

[c] Central Research and Development, HanpoongPharm. Co., LTD., Wanju 54843, Korea

[d] College of Pharmacy, Chungbuk National University, Cheongju, 28160, Korea

[e] Biotechnology Process Engineering Center, Korea Research Institute of Bioscience and Biotechnology, Cheongju, 28116, Korea

Table of Contents

Fig. S1. HRESIMS spectrum of compound **1**........ 3

Fig. S2. ^{1}H NMR spectrum of compound **1** in DMSO-d_6 (700 MHz)........ 4

Fig. S3. ^{13}C NMR spectrum of compound **1** in DMSO-d_6 (175 MHz)........ 5

Fig. S4. ^{1}H-^{13}C HSQC NMR spectrum of compound **1** in DMSO-d_6 (700 MHz)........ 6

Fig. S5. COSY NMR spectrum of compound **1** in DMSO-d_6 (700 MHz)........ 7

Fig. S6. HMBC NMR spectrum of compound **1** in DMSO-d_6 (700 MHz)........ 8

Fig. S7. ROESY NMR spectrum of compound **1** in DMSO-d_6 (700 MHz)........ 9

Fig. S8. IR spectrum of compound **1**........ 10

Fig. S9. HRESIMS spectrum of compound **2**........ 11

Fig. S10. ^{1}H NMR spectrum of compound **2** in DMSO-d_6 (700 MHz)........ 12

Fig. S11. ^{13}C NMR spectrum of compound **2** in DMSO-d_6 (175 MHz)........ 13

Fig. S12. ^{1}H-^{13}C HSQC NMR spectrum of compound **2** in DMSO-d_6 (700 MHz)........ 14

Fig. S13. COSY NMR spectrum of compound **2** in DMSO-d_6 (700 MHz)........ 15

Fig. S14. HMBC NMR spectrum of compound **2** in DMSO-d_6 (700 MHz)........ 16

Fig. S15. ROESY NMR spectrum of compound **2** in DMSO-d_6 (700 MHz)........ 17

Fig. S16. IR spectrum of compound **2**........ 18

Fig. S17. Effects of compounds **1** and **2** on cell viability........ 19

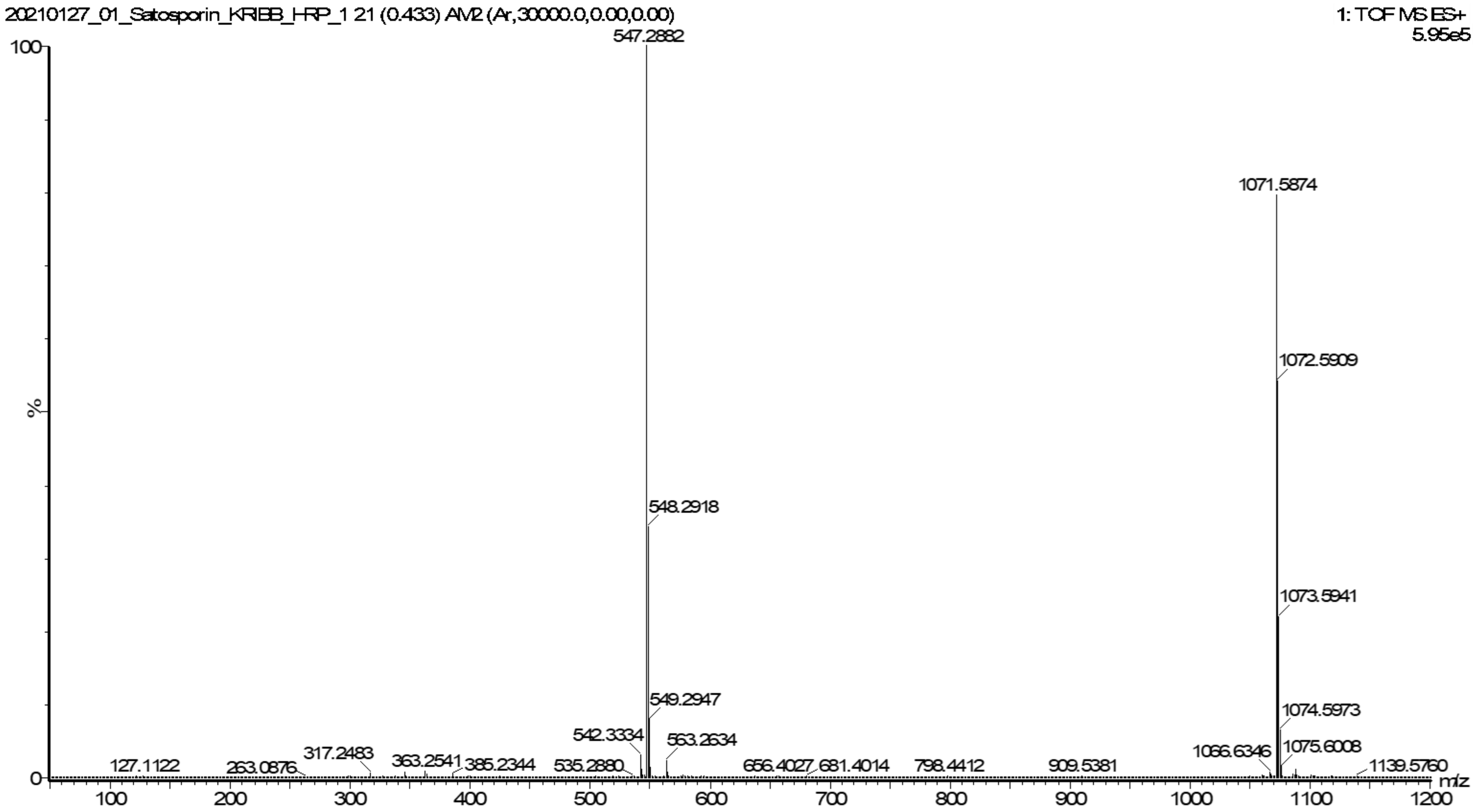

Fig. S1. HR-ESI-TOF-MS positive spectrum in compound **1**

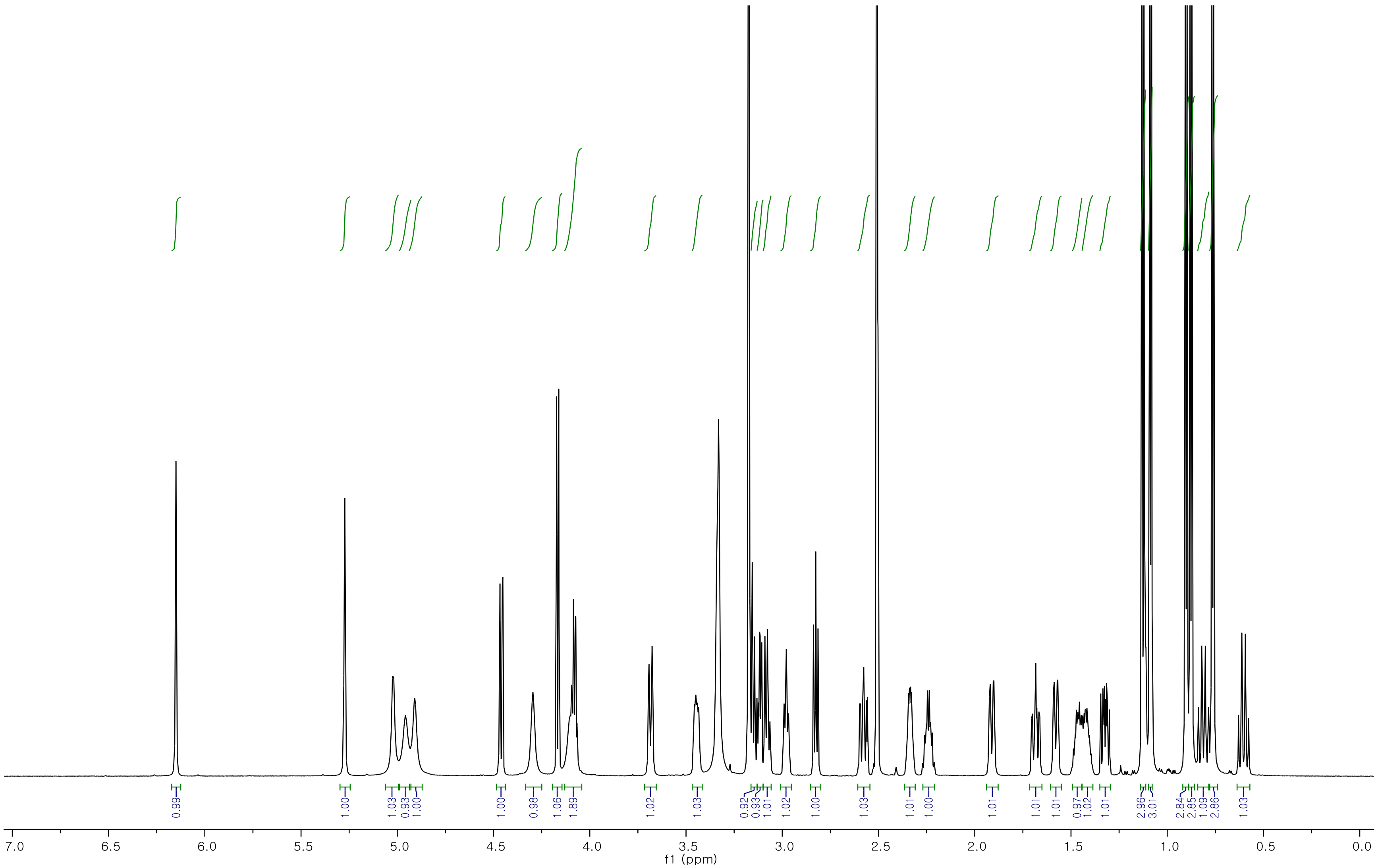

Fig. S2. ^{1}H NMR spectrum of compound **1** in DMSO-d_6 (700 MHz)

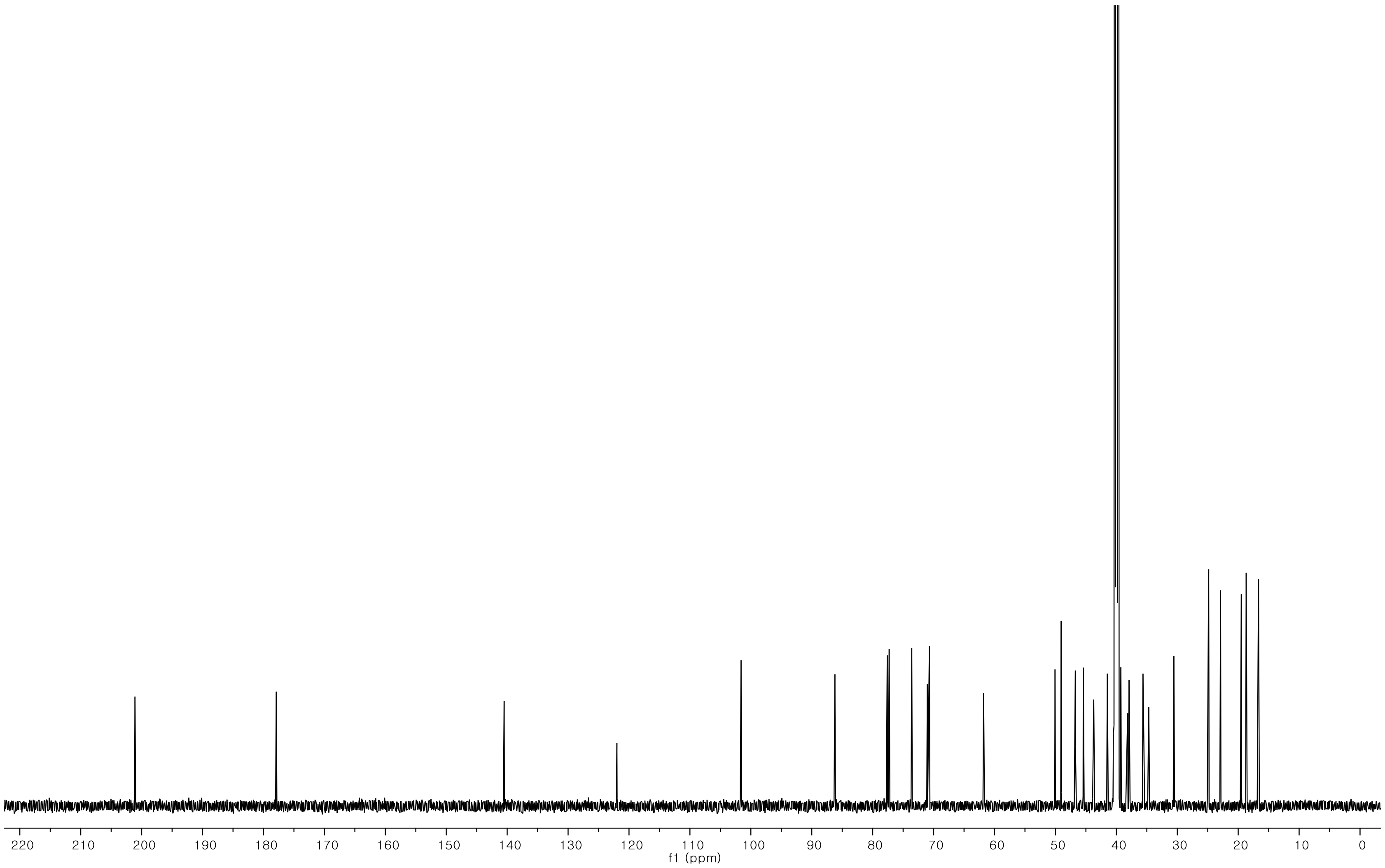

Fig. S3. ^{13}C NMR spectrum of compound **1** in DMSO-d_6 (175 MHz)

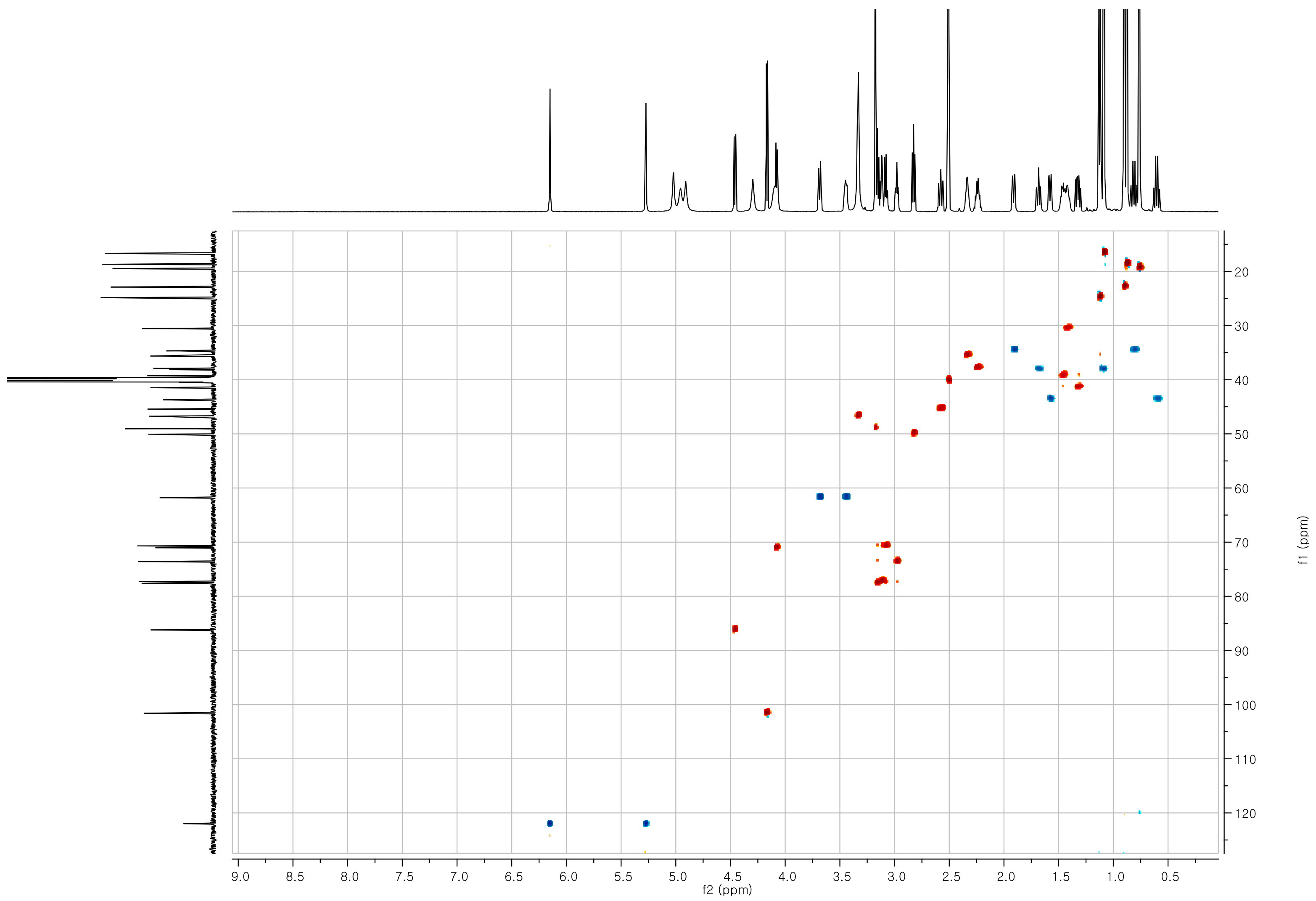

Fig. S4. ^{1}H-^{13}C HSQC NMR spectrum of compound **1** in DMSO-d_6 (700 MHz)

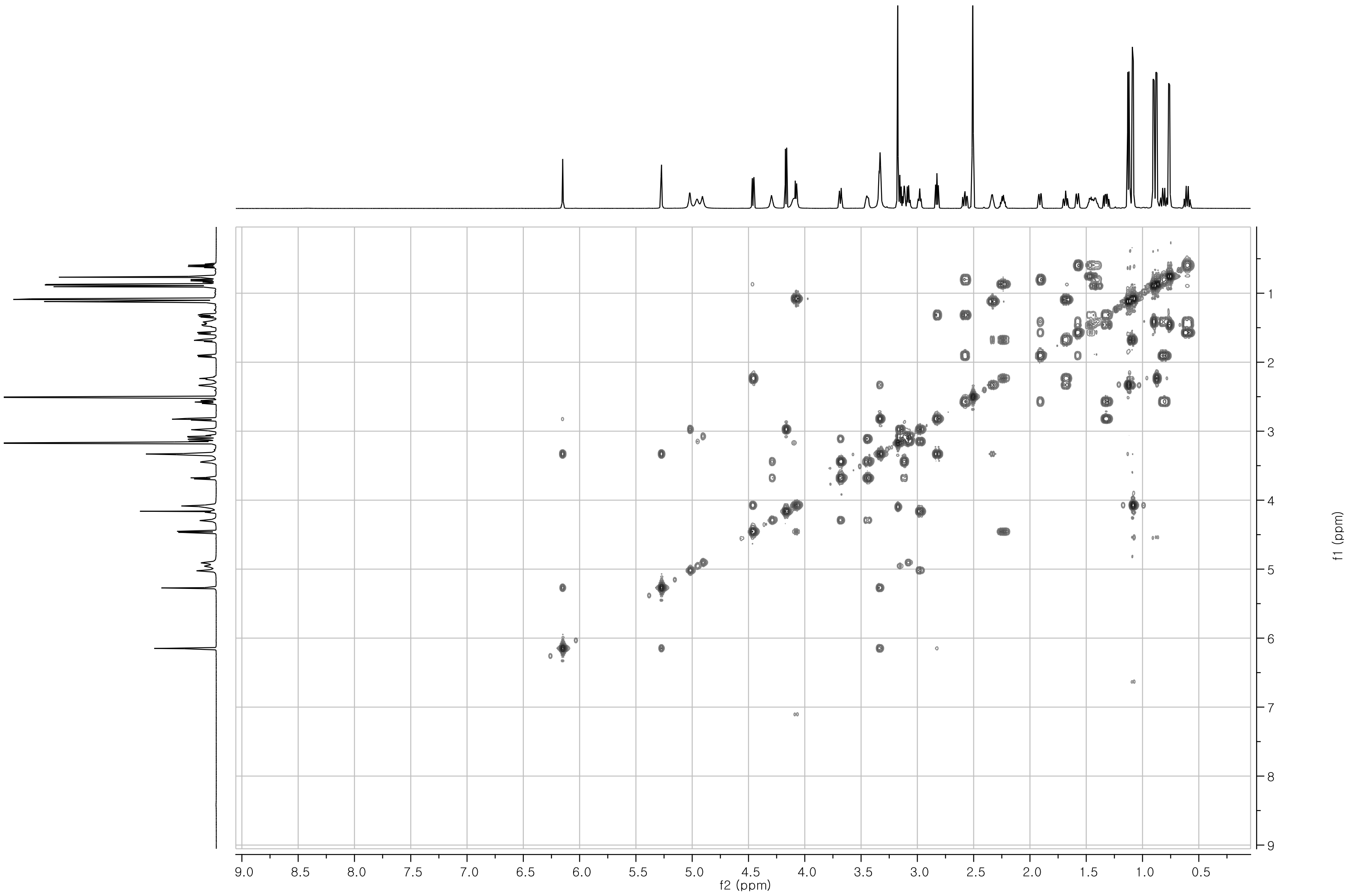

Fig. S5. COSY NMR spectrum of compound **1** in DMSO-d_6 (700 MHz)

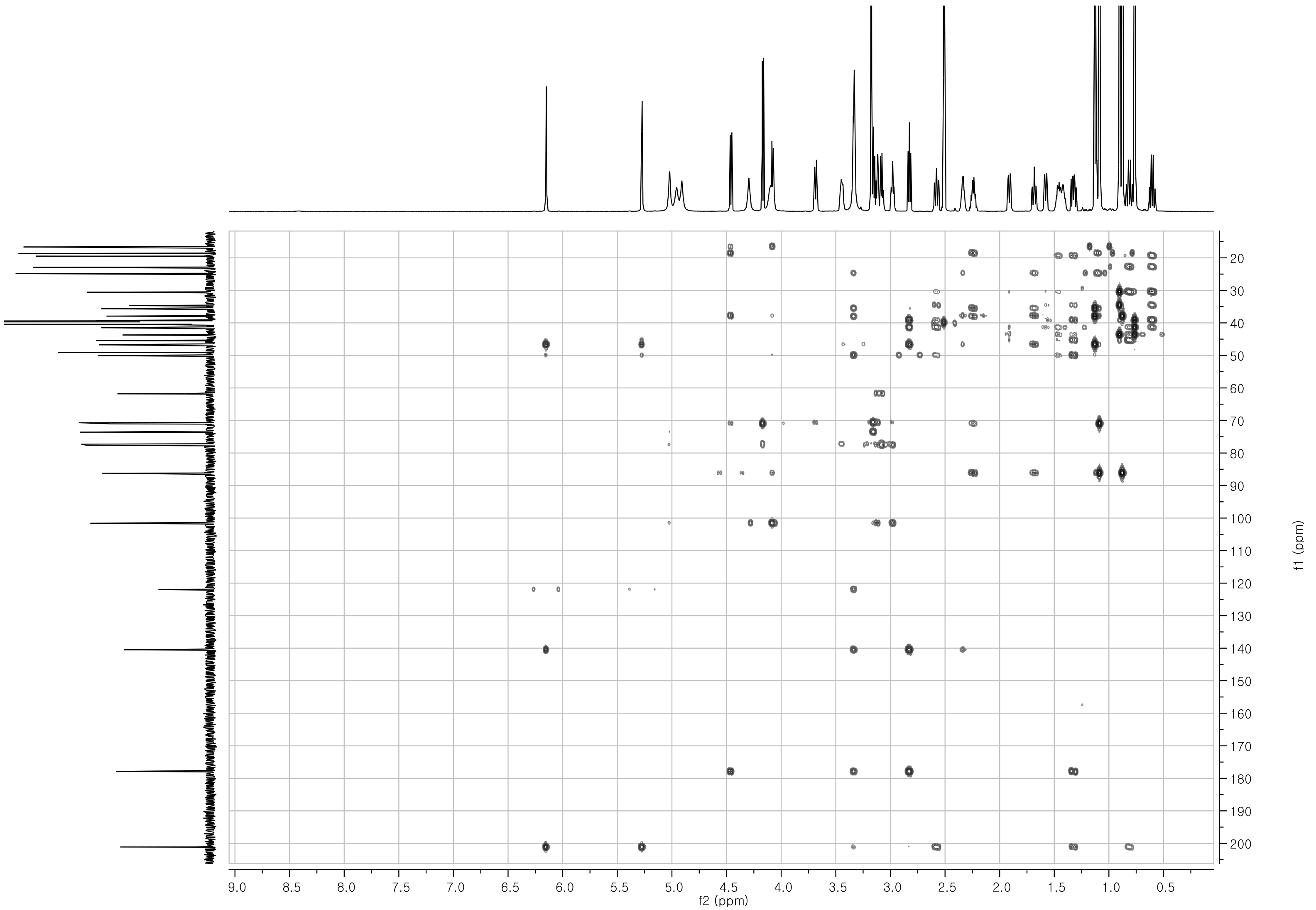

Fig. S6. HMBC NMR spectrum of compound **1** in DMSO-d_6 (700 MHz)

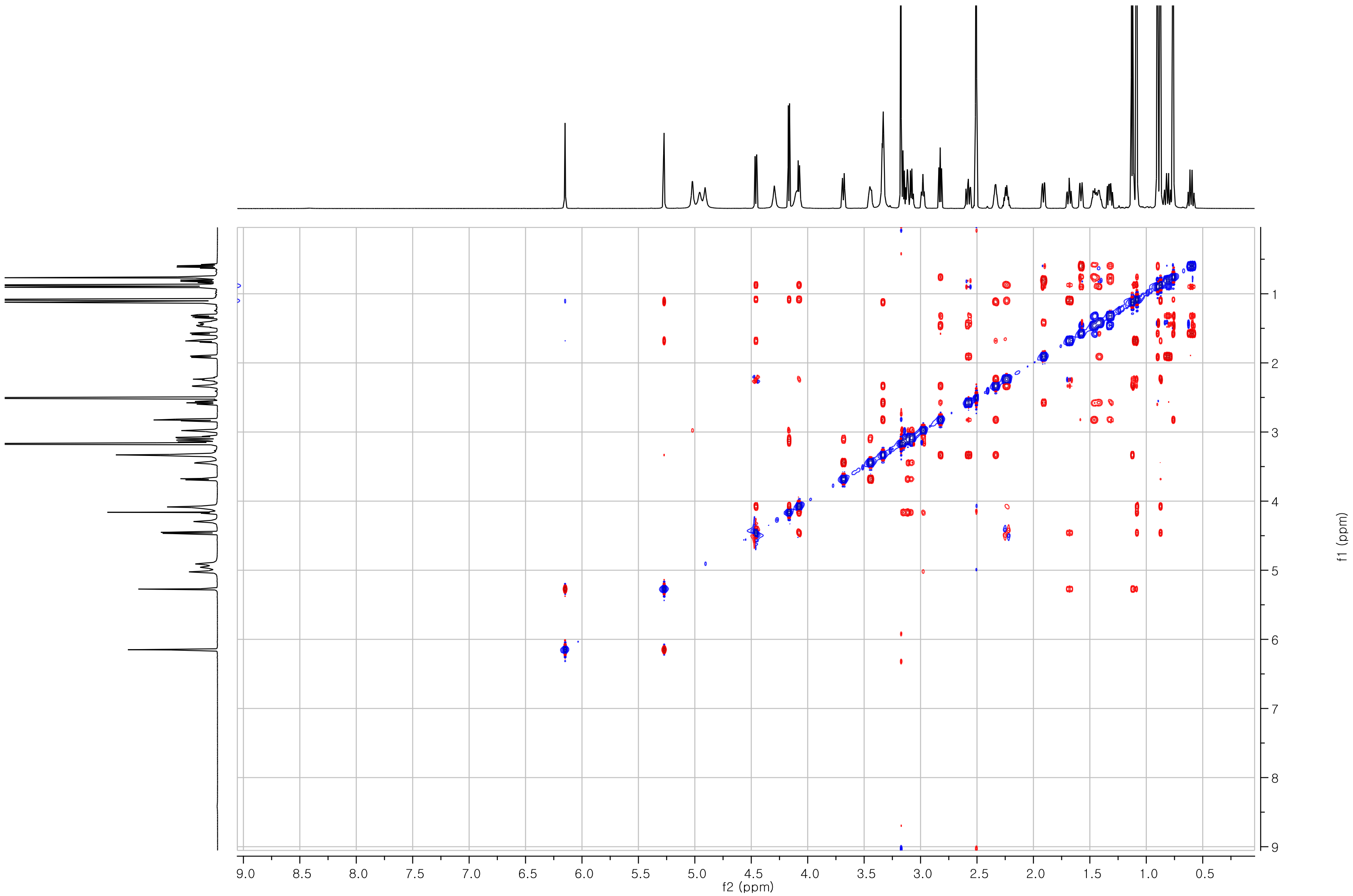

Fig. S7. ROESY NMR spectrum of compound **1** in DMSO-d_6 (700 MHz)

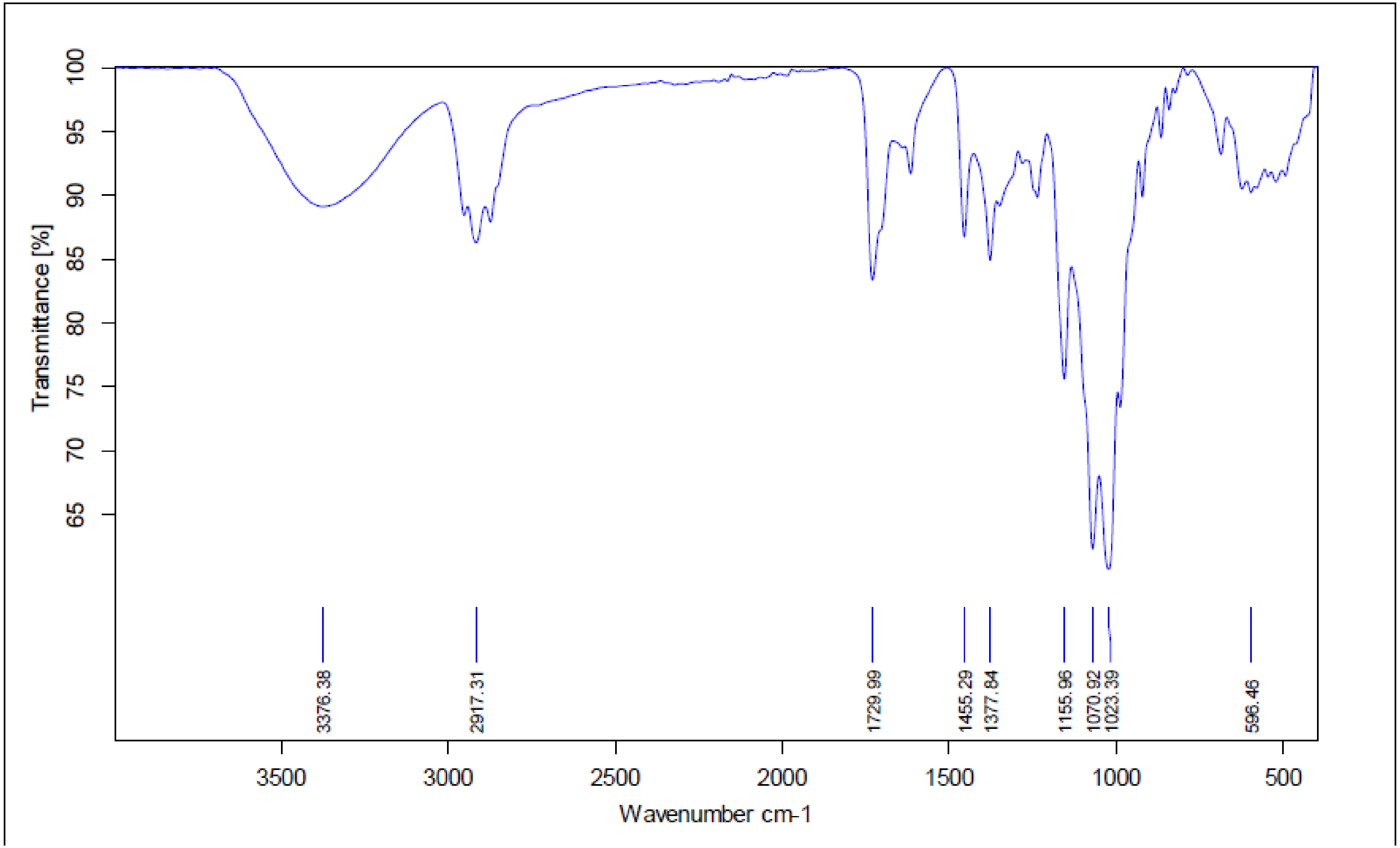

Fig. S8. IR spectrum of compound **1**

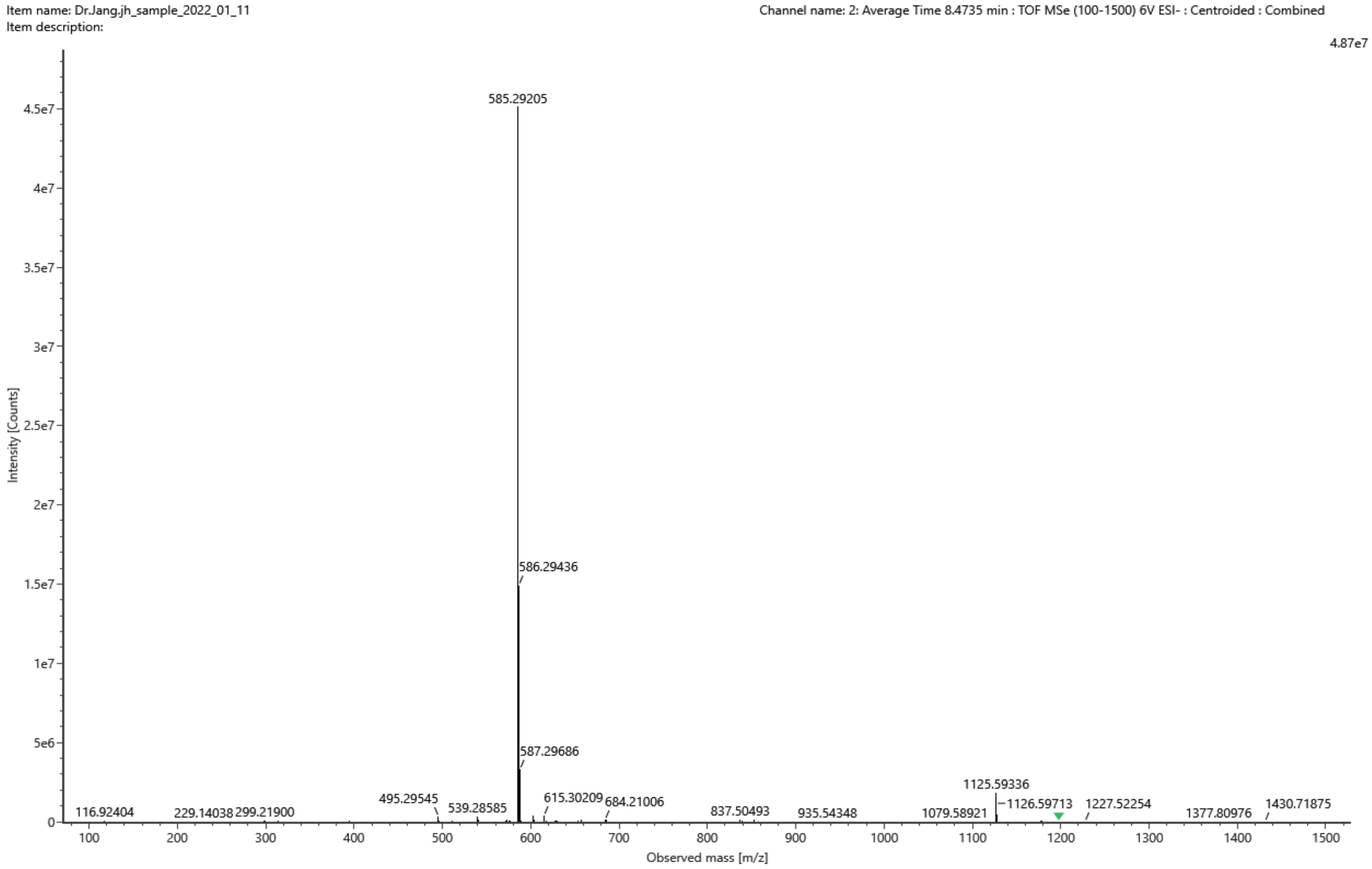

Fig. S9. HR-ESI-QTOF-MS positive spectrum in compound **2**

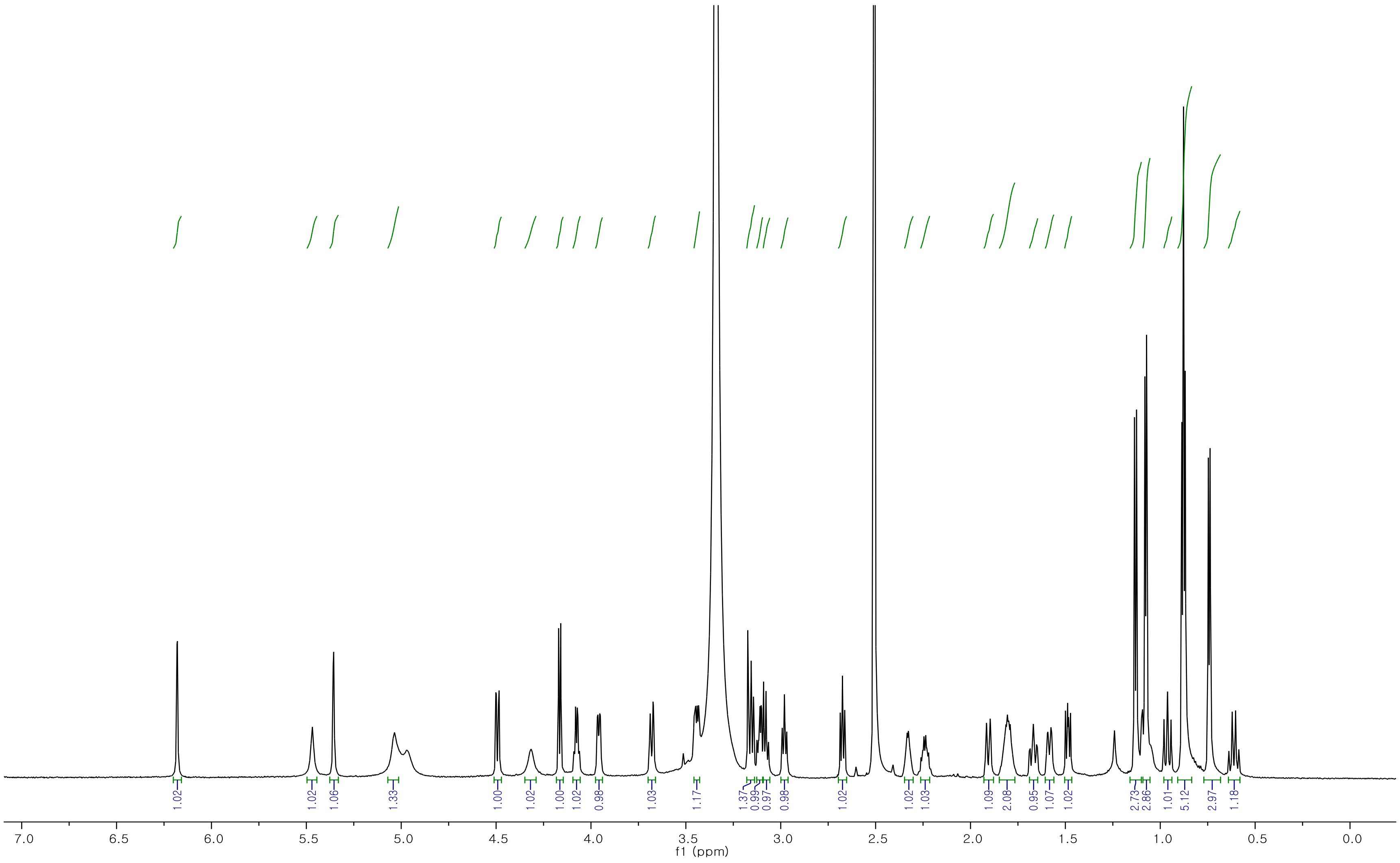

Fig. S10. ^{1}H NMR spectrum of compound **2** in DMSO-d_6 (700 MHz)

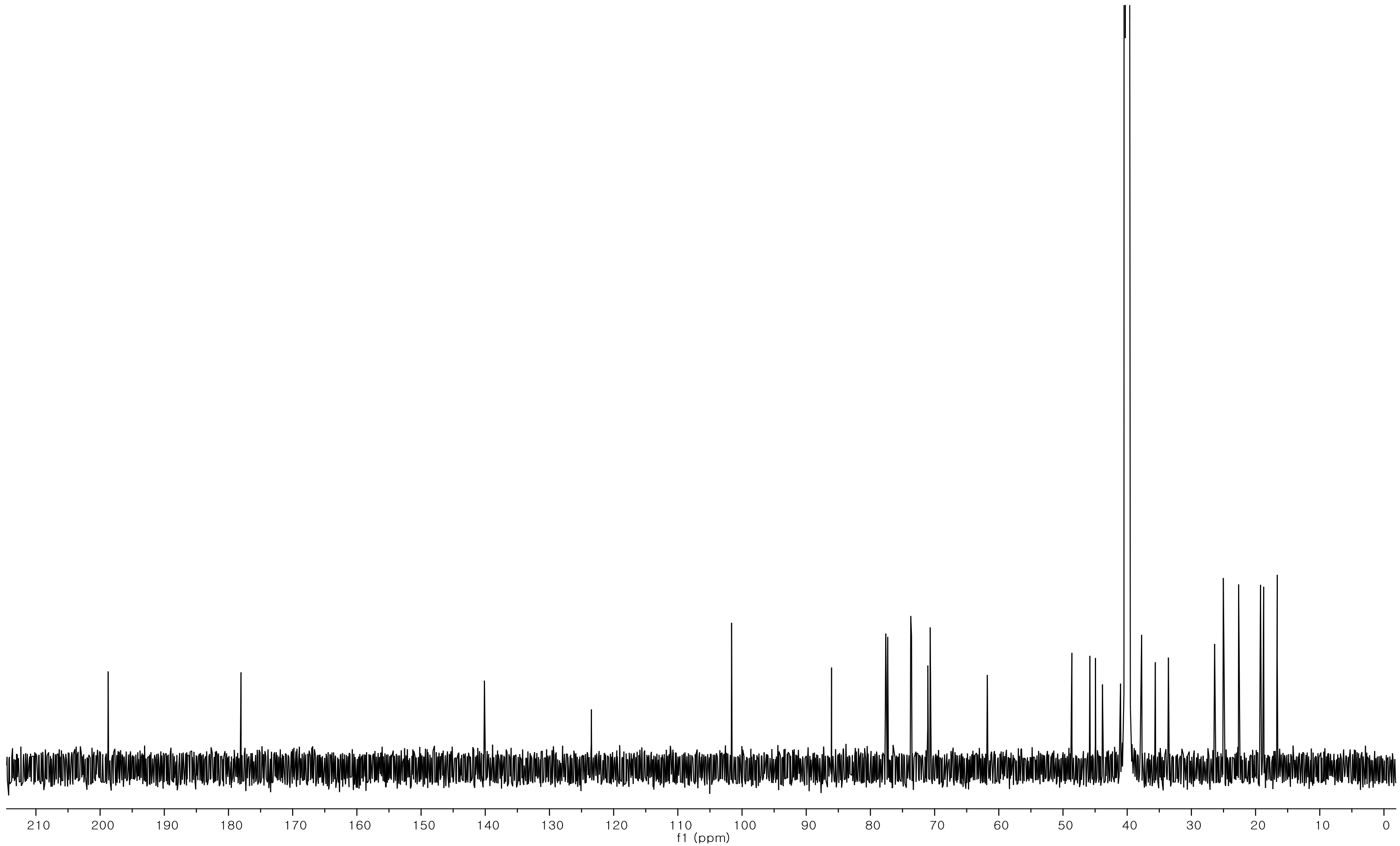

Fig. S11. ^{13}C NMR spectrum of compound **2** in DMSO-d_6 (175 MHz)

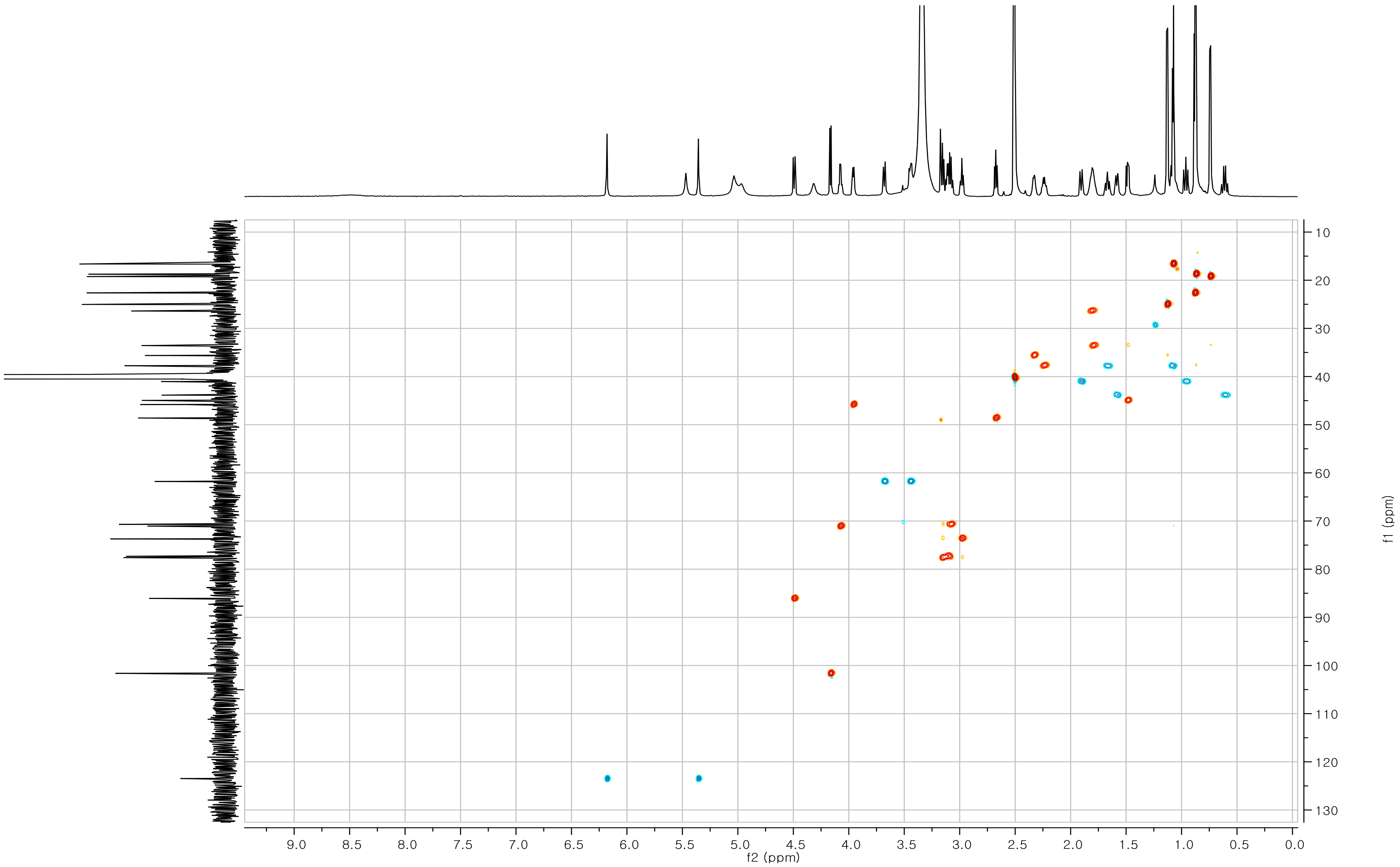

Fig. S12. ^{1}H-^{13}C HSQC NMR spectrum of compound **2** in DMSO-d_6 (700 MHz)

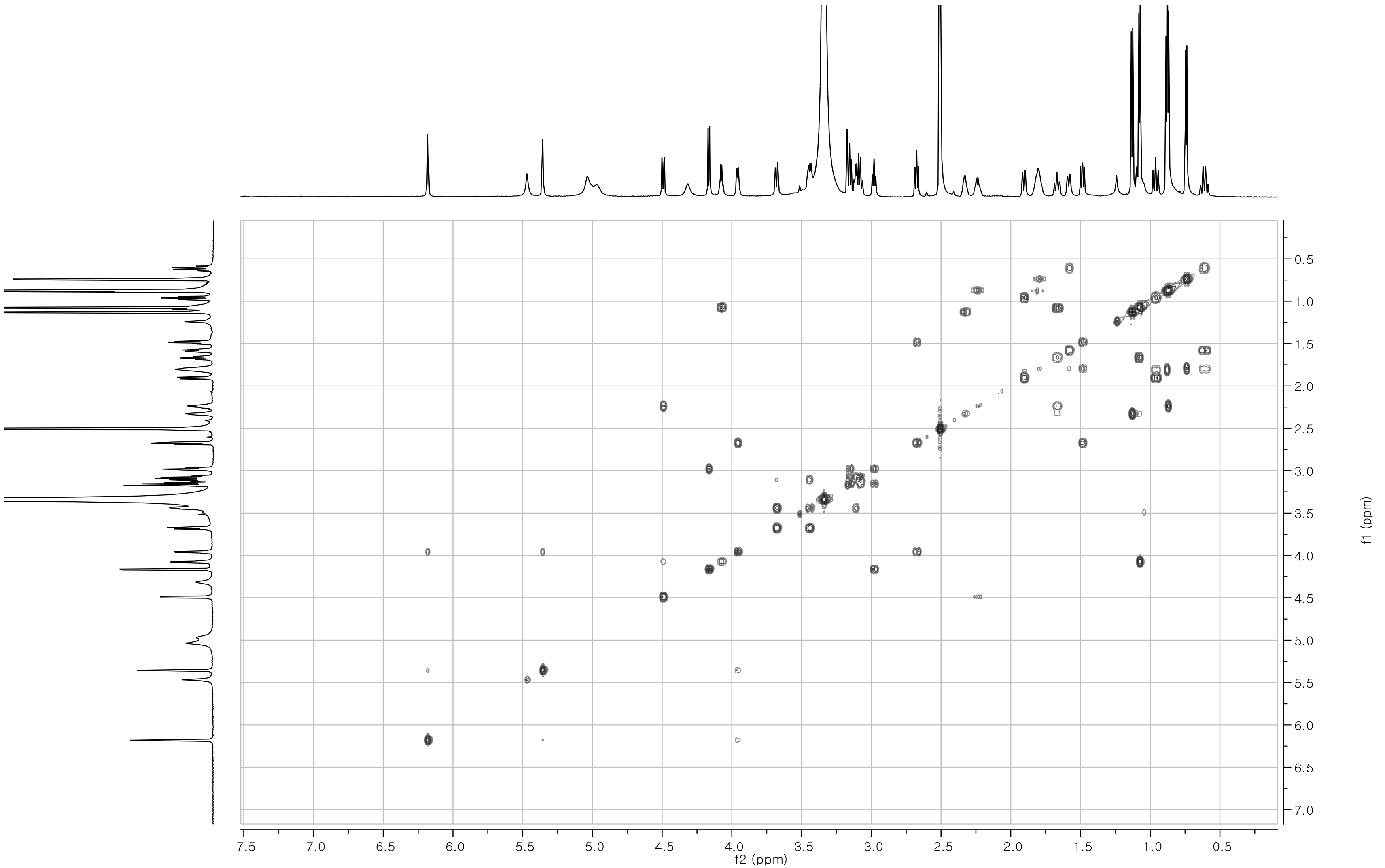

Fig. S13. COSY NMR spectrum of compound **2** in DMSO-d_6 (700 MHz)

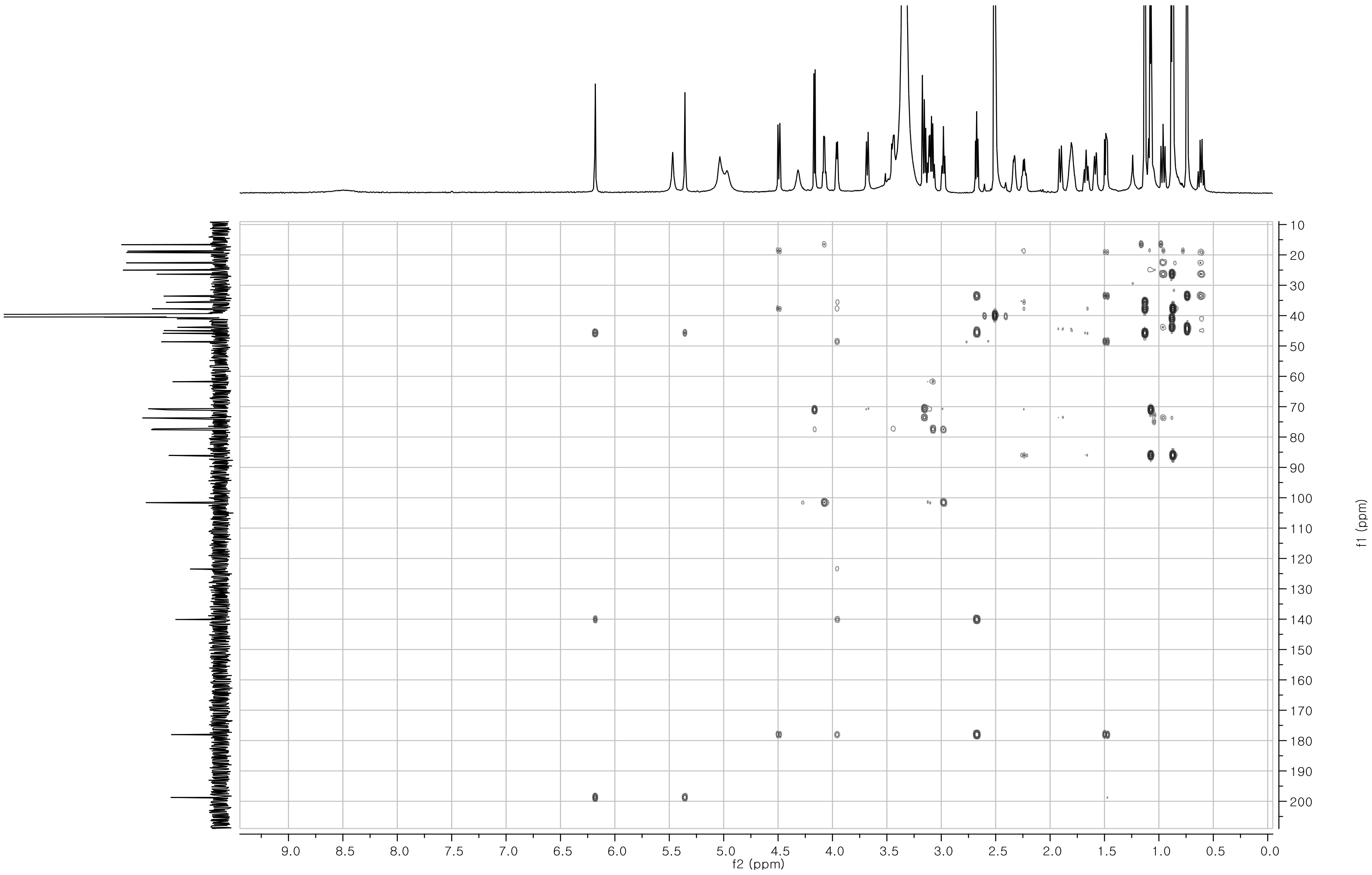

Fig. S14. HMBC NMR spectrum of compound **2** in DMSO-d_6 (700 MHz)

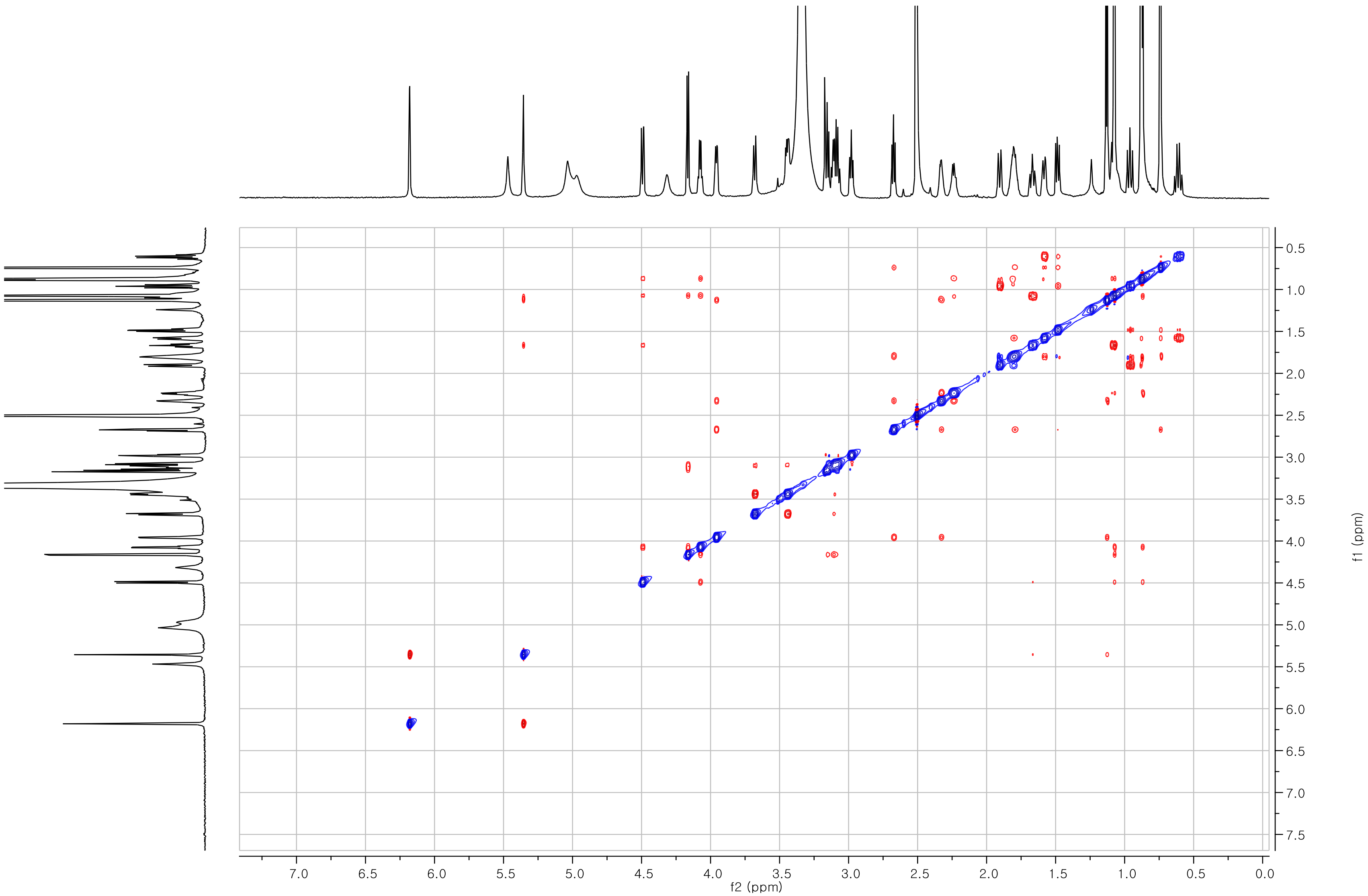

Fig. S15. ROESY NMR spectrum of compound **2** in DMSO-d_6 (700 MHz)

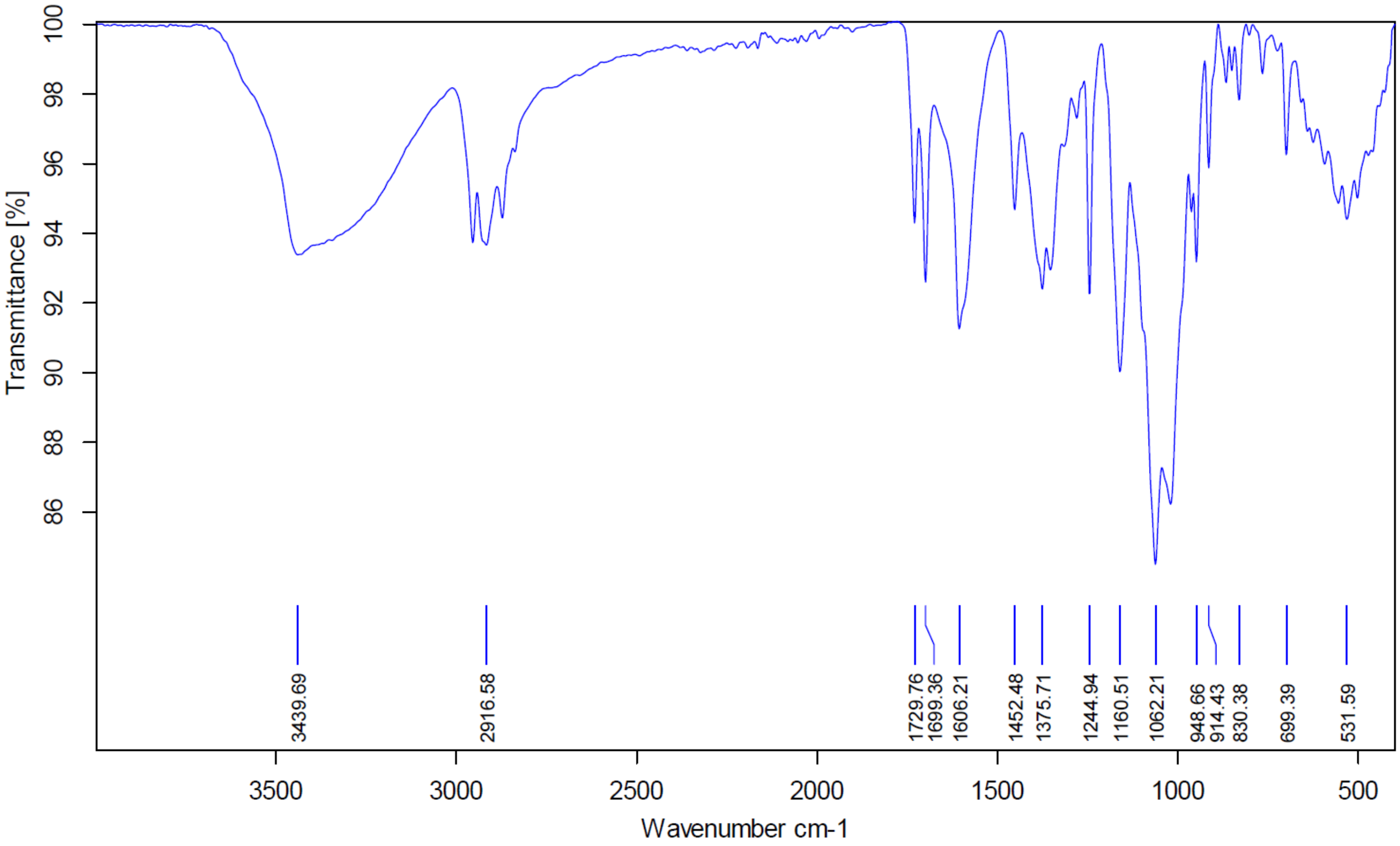

Fig. S16. IR spectrum of compound **2**

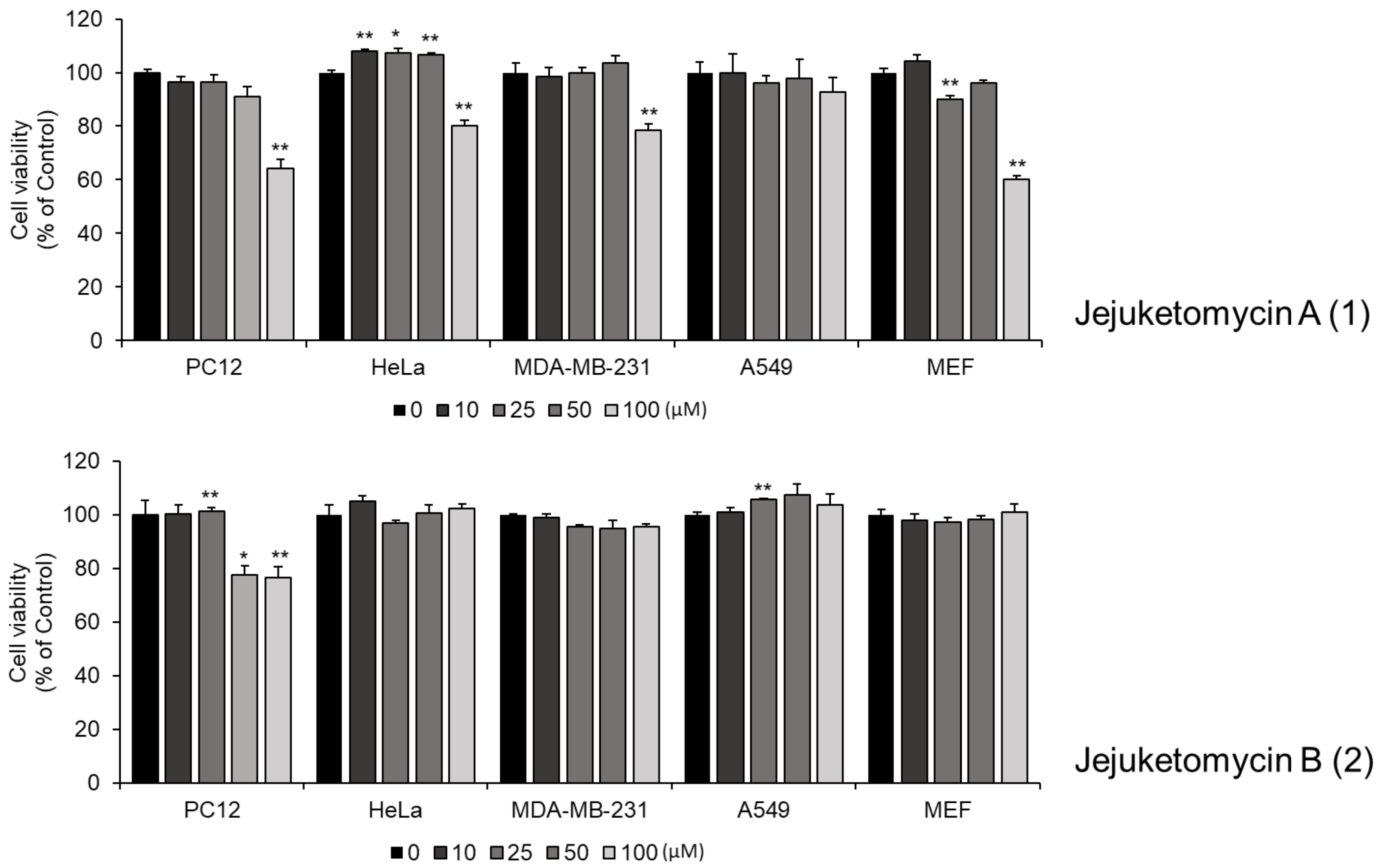

Fig. S17. Effects of **1** and **2** on cell viability. Cells were seeded on a 96-well plate and treated with indicated concentrations of **1** and 2 for 24 h. Cell viability was measured using the EZ-Cytox colorimetric assay kit (mean ± SD, n = 3).
